# Comment on: “Mitochondrial Mechanisms of Neuromuscular Junction Degeneration with Aging. *Cells* 2020, *9*, 197”

**DOI:** 10.3390/cells9081796

**Published:** 2020-07-29

**Authors:** Allen Herbst, Judd M. Aiken, Debbie McKenzie, Jonathan Wanagat

**Affiliations:** 1 Department of Agricultural, Food and Nutritional Sciences, University of Alberta, Edmonton, AB T6G 2R3, Canada; judd.aiken@ualberta.ca; 2 Department of Biological Sciences, University of Alberta, Edmonton, AB T6G 2R3, Canada; debbie.mckenzie@ualberta.ca; 3 Veterans Administration Greater Los Angeles Healthcare System, Los Angeles, CA 90095, USA; JWanagat@mednet.ucla.edu; 4 Division of Geriatrics, Department of Medicine, UCLA, Los Angeles, CA 90095, USA

“The main conclusions are that the ageing atrophy begins as early as around 25 years of age and thereafter accelerates and, for this muscle, is caused mainly by a loss of fibers and to a lesser extent by a reduction in fiber size [1].”

Lexell, Taylor, and Sjöström, 1988

In their manuscript, Anagnostou and Hepple declare, “it may be time to retire the idea that mtDNA mutation accumulation in muscle is causally related to atrophy [2].” The authors conflate the global reductions in fiber size with the segmental intrafiber atrophy caused by mtDNA deletions that leads to fiber loss. The “ageing atrophy” described by Lexell et al. encompasses two distinct processes (i.e., the loss of fibers and reductions in fiber size). We operationally define intrafiber atrophy as segments of fibers with a cross-sectional area (CSA) ratio <0.5. The CSA ratio is determined by measuring the area of single muscle fibers at 100um intervals along 1 mm of fiber length. The minimum cross-sectional area is then divided by the average CSA of that same fiber [3,4]. The longest electron transport chain (ETC)-deficient fiber segments have the highest accumulations of mtDNA deletion mutations, exhibit intrafiber atrophy, and activate cell death [3,4,5]. Other ETC-deficient fibers with short segments have not yet expanded sufficiently to activate cell death pathways and thus do not exhibit intrafiber atrophy. Comparing the segmental intrafiber atrophy to global fiber atrophy is inappropriate, as the cellular mechanism(s) and outcomes are different. Intracellular mtDNA deletion mutation accumulation, the ablation of ETC activity, and intrafiber atrophy are parts of a process that results in fiber loss, not global fiber atrophy. 80% of the muscle fibers undergoing cell death in aged rats contain focal accumulations of mtDNA deletion mutations [5]. Further, when mtDNA deletion mutations were experimentally increased in aged rats, the fiber loss increased [6]. The review by Anagnostou and Hepple misinterprets our data, showing that only 5% of Cox-deficient fibers are atrophic. They suggest that the other 95% of Cox-deficient fibers in aged rats will not become atrophic, activate apoptosis, become necrotic, and undergo fiber loss. Their interpretation ignores the basic premise of our model [5] that they accurately summarized, “The prevailing hypothesis put forward is that dysfunctional mitochondria expand along the length of the muscle fiber, resulting in impairment of normal cellular homeostasis, increased oxidative damage, and activation of apoptotic and necrotic cell death pathways that precipitates *segmental* (our clarification added) atrophy and loss of the muscle fiber.” According to this model, at the time of histological examination, each Cox-negative fiber is at a different point in this process. Our model predicts that all Cox-negative, non-atrophic fibers in aged rats will eventually develop segmental atrophy and die. ETC-deficient fibers undergoing segmental intrafiber atrophy, breakage, and death have been caught in the act [7].

Anagnostou and Hepple reference work from their laboratory which found that less than 1% of the gastrocnemius fibers in a single histochemically stained section were cytochrome c oxidase-deficient [8]. Curiously, they used an unconventional dual histochemical approach to detect ETC-deficient fibers, wherein the succinate dehydrogenase (SDH) stain was performed prior to a cytochrome c oxidase (COX) stain. Conventionally, COX is stained prior to SDH [9,10,11,12]. As others have noted, the dual stain must be interpreted cautiously [10]. Even if the dual staining approach used is accurate, a single histological section cannot quantitate the number of events in a volume of tissue [13]. Volume density measurements, which account for the length of these segments, suggest the steady-state abundance of muscle fibers harboring an ETC deficient segment approached 15% in 38-month old rat quadriceps muscle. The frequency of deletion mutations and ETC-deficient fibers increase exponentially with age [14]. In the human vastus lateralis, the fiber number decreases ~50% between 50 and 80 years of age, a number that translates to the loss of 25 fibers/day (0.008%) [15]. The relatively slow progression of fiber loss distinguishes this aging process from disease processes. The cumulative loss of cells in muscle and other tissues irreversibly contributes to the aging process.

Anagnostou and Hepple state, “patients with primary mitochondrial disease have much higher burdens of mtDNA mutation than seen with normal aging, yet their primary muscle phenotype is one of severe exercise intolerance and weakness rather than atrophy.” Neither of the references [16,17] used to support this statement provides data on muscle atrophy. Muscle atrophy is a feature of mitochondrial diseases. In subjects with mitochondrial disease, the body mass index, fat free mass index, skeletal muscle mass index, and appendicular muscle mass index (APMI) were significantly lower than in healthy controls, and the lower APMI was correlated with increased disease severity [18]. Magnetic resonance imaging of the thigh found decreased muscle size, fat infiltration, variable fiber size, and necrosis in mitochondrial myopathy patients [19,20]. Extraocular muscles (EOMs) are particularly vulnerable to ETC-deficient fibers arising in mitochondrial disease [21] and also with age [22] as compared with other post-mitotic tissues. In addition, the genetic induction of mtDNA deletion mutations clearly induces progeroid phenotypes, including decreased muscle mass, in mice [23,24]. The suggestion by Anagnostou and Hepple that deletions are not sufficient to contribute to aging ignores the devastating consequences of deletions on the lifespan [25,26].

We remain inspired by the data collected by Lexell and colleagues that carefully quantitated the muscle parameters in healthy Swedish men across the lifespan [1]. As such, we have looked for molecular and cellular phenomena that might underlie the loss of cells with age. Studies in rats, rhesus macaques, and humans have identified mitochondrial DNA deletion mutations as contributors to age-induced fiber loss [3,9,27,28]. The etiology of aging is understood to be multifactorial [29] and, in muscle, this likely manifests from mechanisms that induce general fiber atrophy as well as mechanisms that induce fiber loss.
